# The Potential Role for Host Genetic Profiling in Screening for Chlamydia-Associated Tubal Factor Infertility (TFI)—New Perspectives

**DOI:** 10.3390/genes10060410

**Published:** 2019-05-28

**Authors:** Jelena Malogajski, Ivan Branković, Jolande A. Land, Pierre P. M. Thomas, Servaas A. Morré, Elena Ambrosino

**Affiliations:** 1 Institute of Public Health Genomics, Department of Genetics and Cell Biology, Research Institute GROW, Faculty of Health, Medicine & Life Sciences, University of Maastricht, 6211 LK Maastricht, The Netherlands; jelena.malogajski@liu.edu (J.M.); ibrankovic80@gmail.com (I.B.); j.land@maastrichtuniversity.nl (J.A.L.); j.land@maastrichtuniversity.nl (P.P.M.T.); samorretravel@yahoo.co.uk (S.A.M.); 2 Department of Public Health, School of Health Professions, Long Island University–Brooklyn, Brooklyn, New York, NY 11201, USA; 3 Department of Molecular Biology, Max Planck Institute for Infection Biology, 10117 Berlin, Germany; 4 Laboratory of Immunogenetics, Department of Medical Microbiology and Infection Control, VU University Medical Center, 1081 HV Amsterdam, The Netherlands

**Keywords:** *Chlamydia trachomatis*, tubal factor infertility (TFI), screening, diagnostic test, host genetic markers

## Abstract

Host immunogenetic factors can affect late complications of urogenital infections with *Chlamydia trachomatis*. These findings are creating new avenues for updating existing risk prediction models for *C. trachomatis*-associated tubal factor infertility (TFI). Research into host factors and its utilization may therefore have future implications for diagnosing *C. trachomatis*-induced infertility. We outline the epidemiological situation regarding *C. trachomatis* and TFI in high-income countries. Thereupon, we review the main characteristics of the population undergoing fertility work-up and identify screening and diagnostic strategies for TFI currently in place. The Netherlands is an exemplary model for the state of the art in high-income countries. Within the framework of existing clinical approaches, we propose a scenario for the translation of relevant genome-based information into triage of infertile women, with the objective of implementing genetic profiling in the routine investigation of TFI. Furthermore, we describe the state of the art in relevant gene- and single nucleotide polymorphism (SNP) based clinical prediction models and place our perspectives in the context of these applications. We conclude that the introduction of a genetic test of proven validity into the assessment of TFI should help reduce patient burden from invasive and costly examinations by achieving a more precise risk stratification.

## 1. Introduction

Worldwide, 10–15% of couples trying to conceive suffer from infertility [1,2]. In 11–30% of these couples, infertility is related to tubal pathology [1]. *Chlamydia trachomatis* urogenital infection (henceforth in the text referred to as Chlamydial infection) is one of the most common causes of damage to the fallopian tubes and subsequent tubal factor infertility (TFI). Overall, the proportion of TFI attributed to *C. trachomatis* is estimated to be 45% [3]. Such a high percentage stresses the importance of understanding the basic mechanisms behind ascending Chlamydial infections, its complications, and translating the knowledge into healthcare.

Timely diagnosis, treatment, and prevention of late complications in Chlamydia infections are challenging. Up to 90% of infections in women and up to 50% in men are asymptomatic [4,5] and therefore go unnoticed and untreated. Moreover, Chlamydial infections can have very diverse courses in the genital tract. Roughly 45% of asymptomatic women clear the infection within the first year without developing any complications [6]. In others, the bacterium causes persistent infections and sequelae, such as endometritis, salpingitis, pelvic inflammatory disease (PID), ectopic pregnancy, and TFI [5,7,8]. Its urogenital co-infection with human papillomavirus bears additional clinical relevance, as one can heighten the risk of contracting the other, and the two mutually exacerbate the clinical course in afflicted patients [9,10]. Women with Chlamydial infection have a higher risk of developing cervical cancer [11].

A growing body of research exists on associations of Chlamydia host immunogenetic factors with Chlamydia infection outcomes [12,13,14,15,16,17,18]. These genetic variants, or single nucleotide polymorphisms (SNPs), reside in genes coding for various immune response functions [12]. Thus, new insights from research on host immunogenetic factors may have implications for diagnosing Chlamydia-induced infertility, given the fact that the analysis of pertinent SNP-based variants has the potential to improve the accuracy of estimating the risk of TFI. Considering that genetic factors contribute with as much as 39% to the immune response variation in Chlamydial infections (findings based on twin studies of ocular infection [19]), there is a strong argument to be made in favor of including genome-based patient data in clinical assessments of related sequelae.

## 2. The Dutch Experience as the Model for the High-Income World

The Netherlands boasts a comprehensive body of research on complications of urogenital infections with *C. trachomatis*. Additionally, the increasing incidence of Chlamydial infections in the Netherlands is in accordance with general trends in Europe and the United States [20,21]. Furthermore, the general tendency toward delaying motherhood, typical for high-income countries, has been observed over the recent decades in these countries, including the Netherlands [22,23]. Therefore, we argue that the Dutch perspective on the role of host-genetic profiling in screening for urogenital Chlamydia-associated TFI can successfully be extrapolated onto similar societal and healthcare settings. 

Between 2004 and 2012, 10–13% of women visiting sexually transmitted infections (STI) clinics tested positive for Chlamydia. Since 2012, Chlamydia has been the most common bacterial infection registered in Dutch STI clinics [24]. The number of diagnosed cases has been steadily rising since then. In 2015, Chlamydia infection was diagnosed in 13.7% of attendees of STI clinics in the Netherlands, an increase of 1.1% compared to 2014 [25]. In 2016, a more dramatic increase of 11% was observed, compared to 2015 [26]. A comparable increase has been reported Europe-wide in the last decade, which could only partially be explained by a rise in numbers of tests performed and their higher sensitivity [27]. Although the overall trends in the EU/EEA countries have stabilized since 2015, young adult women retain the highest incidence rates [21]. In the USA, rates of reported urogenital Chlamydia cases rose by 4.7% during 2015–2016, whereas the infection rate in women was twice the rate reported in men [20]. These statistics imply a pervasive (or, in the case of the USA, increased) exposure of young women to Chlamydial infection very early in their reproductive years. Given the delayed age of attempting to start a family, a conceivable outcome of this is a potentially higher lifetime exposure to sexually transmitted infections, including Chlamydia, and thereby a higher likelihood of associated pathologies.

The mean maternal age at delivery of the first child has increased from 25.6 years in 1980 to 29.7 in 2016 [28]. In 2009, 4% of children in the Netherlands were born to mothers who were 40 years or older [29]. In 2012, 20% of all births were to first-time mothers older than 35 [30]. In some fertility clinics in the Netherlands, the proportion of women older than 35 years seeking medical care has increased four times in the last 20 years [31]. Female age is the single most important determinant of a couple’s fertility, making it a very important characteristic of the target population [32]. A study conducted in the Netherlands found a strong relation between conception at advanced age and the increased demand for fertility care [31].

There are several challenges in defining the population of women who may potentially benefit from introducing genetic testing into routine clinical investigation of TFI. The exact incidence and prevalence of the Chlamydial infection [33] are unknown, and there is uncertainty about the proportion of women with Chlamydial infections who will over time develop PID and TFI [34]. Several rounds of population screening for Chlamydial infection have been conducted, offering partial insight into the overall prevalence. In the selected regions of Amsterdam, Rotterdam, and several municipalities in South Limburg [35,36], prevalence in young women varied from 2 to 4.2%. A follow-up study evaluating the effectiveness of these screening rounds [37] did not find a significant decrease in positivity rates in any region or socio-demographic group.

There is a lack of prospective studies quantifying the risk of developing TFI over time after an untreated Chlamydial infection. Overall, research offers varying findings, with PID occurring in 2–4.5% to 30% of women with a previously untreated Chlamydial infection, and TFI developing in 10–20% of women with PID [33]. The risk of infertility correlates with a woman’s number of PID episodes [38]. The fact that most women with TFI have not suffered from PID (despite testing positive for *C. trachomatis*) implies that subclinical PID often underlies TFI [39,40]. Studies using mathematical models in their quantification of the risk of TFI after lower genital tract Chlamydial infections also offer different estimates, varying between 0.1 and 5% [41,42]. In 2015, a large longitudinal study into long-term complications of Chlamydial infections, named NECCST, has started in The Netherlands. The study is devised as a continuation of the Chlamydial Screening Implementation study completed in 2011. A large cohort (>10,000) of women who previously tested positive for Chlamydial and negative controls will be followed over a 10-year period. The aim of the study is to quantify the risk of developing PID, ectopic pregnancy, and TFI in women with and without previous Chlamydial infection, as well as to examine how certain genetic and behavioral characteristics affect such risk [43].

**Figure 1:**
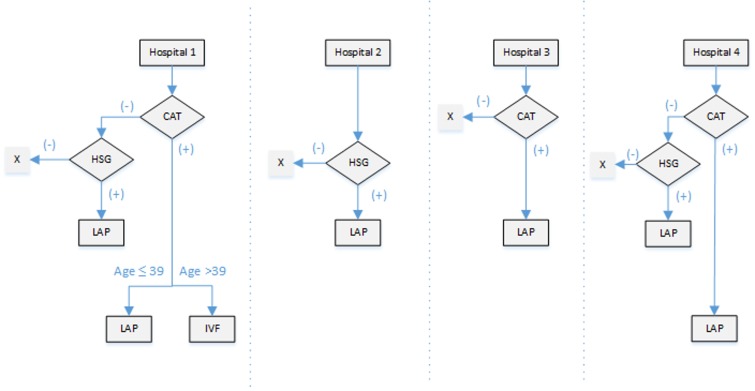
Overview of varying strategies in different fertility clinics across The Netherlands. In Hospital 1, tubal assessment begins with Chlamydia IgG antibody testing (CAT). Whenever CAT is negative (CAT-), hysterosalpingography (HSG) is done. In the case of abnormal HSG, the patient is referred for laparoscopy. In case CAT is positive (CAT+), CAT+ women undergo laparoscopy if younger than 39, whereas older women are directly referred for in vitro fertilization (IVF). In Hospital 2, CAT is not performed. HSG is done as the primary investigation of tubal patency, and laparoscopy is performed only in patients with abnormal HSG results. In Hospital 3, patients are first screened by CAT. In the case of CAT+, laparoscopy is performed. In the case of CAT-, no additional testing is done. HSG as a screening test is totally abandoned. In Hospital 4, screening starts with CAT. CAT+ patients undergo laparoscopy regardless of their age, CAT- patients have HSG and, in the case of an abnormal HSG, they undergo laparoscopy. The symbol X stands for no referral for further diagnostics.

Screening and surveillance data on Chlamydial infection show either persistent or increasing prevalence rates of infection among young adults. This might have clinical implications 10–15 years later, when these women wish to have children. In our investigation of TFI screening strategies in Dutch hospitals (data presented here for the first time), we collected data from three university settings and one general hospital, as representatives for the fertility clinics in the Netherlands. There is currently no consensus on the sequence of diagnostic procedures for tubal function in routine fertility work-up in the Netherlands, due to which diagnostic strategies in Dutch hospitals clearly differ considerably (Figure 1). 

In Dutch hospitals, Chlamydia IgG antibody testing (CAT) in serum is a widely used test in the screening of TFI. The advantage of CAT is that it is easy to perform, inexpensive, non-invasive, and not associated with complications. Anti-Chlamydia IgG antibodies remain present in blood for years after the infection and are considered as markers of previous infections. Nonetheless, the presence of antibodies does not inform us about the course of infection and development of late sequelae [44]. There are a number of studies estimating the accuracy of CAT in screening for TFI [34,45,46]. An early meta-analysis on the predictive value of CAT [45] found that the sensitivity ranged from 21 to 90%, but it has been shown that test accuracy depends on the type of CAT-assay used, C positive (CAT+), the age of the patient will define what the following step is. 

Sensitivity for tubal pathology of the most accurate CAT was assessed at 60%, with a specificity of 85–90% [41]. Research shows that, in 40–50% of women who are testing positive for CAT, no tubal pathology is found with laparoscopy (positive predictive value, PPV), whereas 10–20% of women testing negative do have tubal pathology (negative predictive value, NPV) [46,47,48].

In the Netherlands, hysterosalpingography (HSG) is either performed following CAT or as an independent test of tubal patency. HSG’s sensitivity and specificity are estimated at 53% and 87%, respectively, compared to laparoscopy [49,50]. The main disadvantage of HSG is that it requires radiologic facilities, is a painful procedure for the patient and may be complicated by an ascending infection. Laparoscopy is considered the reference standard in the assessment of tubal function. However, it is an invasive procedure associated with discomfort for the patient, it has to be performed under general anesthesia, and it is associated with potential surgical complications, a post-surgical recovery period and high costs [51].

## 3. Host Genetic Markers May Contribute to Adequately Assessing the Risk of TFI

As mentioned, positive CAT is not predictive of the course of infection. Testing for variations in genes encoding for innate immunity and inflammatory pathways, including tissue scarring, may be able to provide more insight into the risk of developing late sequelae. Since tubal pathology is a multifactorial and polygenic disease, one single SNP typically cannot predict a major risk, so combinations of SNPs in traits are being evaluated for their predictive value. Most SNP variants lead to partial changes in the function of their respective proteins rather than a complete loss (or gain) of function. Both “protective” and “risk” traits for the development of tubal pathology following Chlamydial infection, or other (bacterial) STIs, have been described [15,52] (Table 1).

The intended added value of combining such host genetic assays with CAT would be to improve the PPV for diagnosing TFI, by reducing the number of false positive CAT results and the number of invasive diagnostic procedures performed in CAT-positive women without TFI. Conversely, the combined assay could improve NPV by reducing the number of patients with false negative CAT results, whose TFI diagnosis would otherwise be delayed by postponing or omitting HSG or laparoscopy.

Our hypothetical screening model (Table 2) presents possible clinical outcomes and the strategy to handle female patients in each group, based on the results of their serological and genetic tests. The model is based on CAT, as a marker of previous Chlamydial infection, and on testing for carriership of SNP variants (i.e. genetic markers), which can either increase or reduce the risk of developing TFI. Different combinations of risk and protective genetic markers can either lead to a low or high predisposition to TFI (for simplicity, we will further on refer to the low presence or absence of risk-increasing genetic markers and/or the high presence of protective genetic markers as low genetic risk, and vice versa). In case a patient’s CAT result is negative and her genetic risk is low, the risk of TFI will be deemed as low. The combination of positive CAT and high genetic risk would potentially identify women at the highest risk of tubal pathology. A positive CAT and low genetic risk profile would indicate that, although the patient had a Chlamydial infection in the past, there is a considerable chance for the infection to have been cleared without complications, due to the presence of protective markers (or absence of risk markers). Lastly, when CAT is negative and genetic markers indicate high genetic risk, the risk for TFI is considered intermediate, as TFI can be caused by bacterial STIs other than Chlamydia. In those cases in which the genetic marker test is simultaneously positive for the SNPs that put the patient at high risk of developing TFI and for the SNPs recognized for their protective role, the decision-making will be more complex and will have to rely on additional clinical variables.

## 4. Conclusions and Outlook

One objective of an SNP-based host genetic assay would be to improve the accuracy of first line testing by combining a serologic and genetic approach, to more accurately determine the TFI risk and reduce the number of misdiagnoses. If successfully implemented, the test would lead to improved clinical decision-making in fertility clinics, a more efficient use of resources, and fewer expenses for the healthcare system. There are, however, challenges to be considered when proposing the introduction of genetic testing in the routine fertility work-up. One of them relates to the intricacies that accompany the implementation of new healthcare technologies. Carriership of SNPs that both increase and reduce the risk of TFI would lead to a “diagnostic grey zone,” making clinicians’ decision processes more complex and hence necessitating well-defined, robust algorithms to aid decision-making. The cases of positive CAT and low genetic risk would also lead to ambiguity and would require active decision-making by specialists. Nevertheless, the model is expected to lead to an overall reduction of the work burden for the involved health workers. Rising costs of healthcare are urging cost-effectiveness of all new diagnostic and treatment strategies. Therefore, there is a need for a comprehensive analysis comparing costs and outcomes of the course of action proposed in our study with the existing combinations of diagnostic strategies currently performed in high-income nations (CAT, HSG, and laparoscopy). Finally, advancements in genomics are driving changes in diagnostic and treatment strategies in many fields of medicine. The main foreseen challenge is creating a low-cost test with straightforward results that are easy to interpret and explain to patients. 

Genetic testing may undeservedly be labeled as high-priced. A recent study reviewing the entire spectrum of economic evaluations associated with genetic tests used for guidance treatments and interventions found no evidence that these tests are either inferior or superior in terms of cost-effectiveness to other medical interventions. They found that how a genetic test is used rather than whether it was used is what had actual economic significance [55]. 

One of the key elements for the implementation of genetic applications in clinical care is its proof of applicability and accuracy [56]. Therefore, successful validation of such tests is paramount. Clinical studies need to be performed in order to demonstrate the benefits of the proposed strategies and to confirm that the introduction of the host genetic markers as an addition to CAT in testing of TFI will result in better patient management and improved clinical outcomes. Another challenge for the healthcare professionals will be to adjust to the new developments. When introducing a genome-based companion diagnostic in the routine fertility work-up, setting up continual education courses for residents and reproductive specialists in order to increase their familiarity with clinical genetics would be an important implementation step. The previous study of Malogajski et al. investigated the attitudes of fertility specialists towards the addition of genetic testing in screening of TFI and on the factors they perceive as barriers and facilitators for the introduction of genetic testing in the routine screening for tubal pathology [57]. They reported additional genetic and genomic training as the leading facilitator. Continual genomic courses for residents and various profiles of fertility specialists designed to increase their familiarity with clinical genetics were recognized by the absolute majority of participants as one of the most important factors in their acceptance of genetic testing in routine fertility work-up.

Moreover, assessing the introduction of similar technologies gives insight into potential outcomes. Sanders and colleagues managed to—albeit modestly—increase the PPV of a post-meningitis hearing loss prediction model for children by adding a palette of SNPs in innate immunity genes [58]. Genetic risk models typically do not outperform clinical models, but what must be taken into consideration is the significance of well-defined study populations and proper stratification, which is frequently lacking in these studies [59,60]. The prediction rule of the SNPs for the TFI host genetic assay will be finalized in a cohort of 1000 women with subfertility and a second confirmation cohort of a comparable size. The assay is to be first implemented in a trail outpatient clinic in the Netherlands in 2020.

However, despite the sizeable component of genetic variation within the overall risk for susceptibility to Chlamydia, our advocacy is not for the use of genetic risk profiles as the single prediction tool in clinical TFI assessment. Piloted prediction models for other diseases [61] indicate the need for including a more comprehensive palette of clinical data, in which the genome-based information further enhances predictive abilities. We believe that such validated combination of clinical and genome-based parameters would allow for an improved triage of female patients suffering from infertility.

## Figures and Tables

**Table 1:** Description and statistics for selected single nucleotide polymorphisms (SNPs) found to be associated with increased risk or protective effect in Chlamydia-associated tubal factor infertility (TFI). The first two results are based on research performed on Dutch female patient cohorts, whereas the third represents a combined odds ratio (OR) and confidence intervals (CI) in joined Dutch and Finnish cohorts.

SNP	Consequence	OR (95% CI)	Reference
*NOD1* + 32656 T > GG	Increased risk of TFI	2.3 (1.1–4.7)	[18]
*TLR2* + 2477 G > A (rs5743708)	Increased risk of TFI	17.5 (0.9–343.0)	[53]
*CXCR5* + 10950 T > C (rs3922)	Protective effect against TFI	0.1 (0.04–0.59)	[54]

**Table 2:** Proposed hypothetical screening model for tubal factor infertility (TFI) based on results of the combined serological Chlamydia IgG antibody test (CAT) and genetic marker (SNP) test, and recommendations for additional testing by hysterosalpingography (HSG) or laparoscopy (LS). CAT and the host genetic marker assay are both non-invasive tests for which a small quantity of blood is needed. Combining CAT and genetic marker test in the initial phases of the infertility investigation may allow for assessing the risk of tubal pathology at an early stage.

CAT Testing	Genetic Markers	Risk Estimate for TFI	Clinical Decisions
Negative	Absence of risk factors and/or presence of protective factors.	Very low	No additional testing required.
Positive	Absence of risk factors and/or presence of protective factors.	Low to intermediate	Past infection, but most likely cleared without complications. To rule out TFI HSG or LS could be considered.
Negative	Presence of risk factors and/or absence of protective factors.	Intermediate	Further investigation by HSG or LS advised, as TFI can be caused by other sexually transmitted infections (STIs).
Positive	Presence of risk factors and/or absence of protective factors.	High	Confirm TFI by LS or refer for in vitro fertilization.
